# Free-Radical Bulk-Photopolymerization Process as a Method of Obtaining Thermally Curable Structural Self-Adhesive Tapes and Effect of Used Type I Photoinitiators

**DOI:** 10.3390/polym12102191

**Published:** 2020-09-24

**Authors:** Konrad Gziut, Agnieszka Kowalczyk, Beata Schmidt

**Affiliations:** Department of Chemical Organic Technology and Polymeric Materials, Faculty of Chemical Technology and Engineering, West Pomeranian University of Technology in Szczecin, 70-322 Szczecin, Poland; konrad.gziut@zut.edu.pl (K.G.); beata.schmidt@zut.edu.pl (B.S.)

**Keywords:** polymer synthesis, bulk photopolymerization, photoinitiators, acrylate syrup, structural adhesives, adhesion

## Abstract

A new fabrication method for thin (120 µm) thermally curable structural self-adhesive tapes (SATs) was demonstrated by utilizing a series of acrylic syrups (ASs) modified using Bisphenol A-based liquid epoxy resin. The acrylic syrups containing poly(butyl acrylate-*co*-butyl methacrylate-*co*-glycidyl methacrylate-*co*-2-hydroxyetyl acrylate-*co*-4-acryloyloxy benzophenone) were synthesized via free-radical bulk-photopolymerization (FRBP) process. Influence of different type I radical photoinitiators (PIs), i.e., α-hydroxyalkylphenones (HPs), acylphosphine oxides (APOs) and its mixtures (HPs/APOs and APO/APO) on selected physico-chemical features of obtained ASs was studied. It turned out that APO-type PIs are more effective in the FRBP process (NMR studies). Self-adhesive tests of SATs revealed that the monomers’ conversion in ASs have a significant influence on adhesion and tack. Moreover, the polymer structures formed at the UV cross-linking stage of SATs significantly affect the cross-linking degree of SATs during thermal curing (differential scanning calorimetry method). The highest values of overlap shear strength were achieved by SATs based on ASs with monomers’ conversion on the level 50–60%.

## 1. Introduction

Structural adhesives in the form of thin films are widely used in automotive and aircraft construction for the fabrication of honeycomb structures because of their form (i.e., thin film), lower joint weight, and uniform distribution of stresses along the joint [1,2,3,4]. The most common type of such adhesives is those intended for thermal curing (i.e., thermally curable structural adhesive in the form of a solid tape). The first structural adhesive tape (SAT) was manufactured by Hexcel from phenolic resin and a polyvinyl formal. Nowadays, several SATs based on different phenolic and epoxy resins are produced [5]. Literature related to the fabrication of adhesive films is limited and the clear information is rather intellectually protected. There are only very few data about structural adhesive films’ preparation or modification. The latest revealed a moisture effect on commercial structural adhesive film [6], using epoxy resins modified with oxazolidinone [7] or polysulfones [8]. A particular kind of the SATs is those with self-adhesive features. They can be prepared, for example, via a UV-photo cross-linking process of reactive acrylate copolymers (obtained in classical free-radical copolymerization process initiated by 2,2′-azobis(isobutyronitryle) and in the presence of organic solvent) compounded with epoxy resin and latent curing agent [9,10,11,12]. Although the developed method of preparation and modification of SATs gives high results of adhesive and mechanical properties, in recent years a very strong emphasis has been placed on waste-free technologies. UV-initiated polymerization fits very well with this global trend. Nowadays, UV technology uses a convenient light sources (light-emitting diodes (LEDs), household lamps, LED bulbs, and the sun) [13] and photopolymerization reactions are encountered in various experimental conditions, i.e., in film [14,15], (micro)heterogeneous media or solid state [16,17], on surface [18,19,20], in ionic liquids [21,22], in situ for the manufacture of microfluidic devices [23,24], and under magnetic field [25]. Relatively rarely the photopolymerization method is used to obtain a liquid polymer or polymer solutions. Admittedly, in the last decade several publications have been published ‘regarding the method of obtaining acrylate syrups via the photoinduced polymerization process that occurs in large amounts of monomers’ mixture and with a mechanical mixing [26,27,28,29,30,31,32]. Obtained products were mainly used as transparent pressure-sensitive adhesives. Nevertheless, the authors did not raise the issue of the impact of photoinitiators (type, quantity) or other factors on the photopolymerization in the bulk (mass) process itself. Type I photoinitiators, especially α-hydroxyketones and acylphosphine derivatives, are widely used in industry for coatings’ forms [33,34], although initiatorless photopolymerization processes are also realized as well [35]. Nevertheless, current research is focusing on new photoinitiators in photopolymerization processes of acrylates, i.e., bis-silyl ketone [36] or novel phosphine oxides [37,38].

The work presented here demonstrates a new, ecological (without solvent), and rapid method of obtaining a photoreactive acrylic resins (acrylic syrups (ASs)) as the main component of structural adhesives in the form of thermally curable structural adhesives’ tapes. The main intention of the authors was to determine the influence of the commercial radical photoinitiator type (α-hydroxyketones, acylphospine oxides and its mixtures) on the polymerization process of (meth)acrylate monomers carried out in large volume of monomers’ mixture and under the influence of mechanical mixing (free-radical bulk-photopolymerization (FRBP) process). The purpose of the work was to demonstrate that the type of radical photoinitiator for the FRBP processes has a key impact on the characteristics of obtained products (i.e., ASs). Moreover, the influence of ASs’ features on the adhesive, mechanical, and thermal properties of the prepared SATs was investigated as well.

## 2. Materials and Methods

### 2.1. Materials

The following components were used for preparation of acrylic syrups: n-butyl acrylate (BA), butyl methacrylate (BMA), 2-hydroxyethyl acrylate (HEA) (BASF, Ludwigshafen, Germany), glycidyl methacrylate (GMA) (Dow Europe GmbH, Horgen, Germany) and 4-acryloyloxy benzophenone (ABP) (Chemitec, Scandiccy, Italy). As radical photoinitiators type I were tested:

(1) α-Hydroxyalkylphenones (HPs):
  - 2-hydroxy-1-(4-(4-(2-hydroxy-2-methylpropionyl)benzyl)phenyl)-2-methylpropan-1-one (Omnirad 127; IGM Resin, Waalwijk, The Netherlands).
  - 1-hydroxycyclohexylphenyl ketone (Omnirad 184, IGM Resins, Waalwijk, The Netherlands).
(2) Acylphosphine oxides (APOs):
  - 2,4,6-trimethylbenzoyl-diphenyl phosphine oxide (Omnirad TPO; IGM Resins, Waalkwijk, The Netherlands).
  - Bis(2,4,6-trimethylbenzoyl)-phenylphosphineoxide (Omnirad 819; IGM Resins, Waalkwijk, The Netherlands).
(3) Mixtures of HPs and APOs:
  - Omnirad 2100 (IGM Resins, Waalkwijk, The Netherlands), a blend of ethyl phenyl(2,4,6-trimethylbenzoyl)phosphinate (ca. 95 wt.%) and phenyl bis(2,4,6-trimethylbenzoyl)-phosphine oxide (ca. 5 wt.%).
  - Omnirad 2022 (IGM Resins, Waalkwijk, The Netherlands), a blend of 2-hydroxy-2-methylpropiophenone (ca. 75 wt.%), phenyl bis(2,4,6-trimethylbenzoyl)- phosphine oxide (ca. 17.5 wt.%) and ethyl phenyl(2,4,6-trimethylbenzoyl)phosphinate (ca. 7.5 wt.%).

The structures of tested radical photoinitiators are presented in Table 1. The monomers and initiators were applied without purification.

The thermally curable double-sided structural self-adhesive tapes (SATs) were compounded using the AS, the Bisphenol A-based liquid epoxy resin with epoxy equivalent weight of ca. 202 g/equiv. and viscosity 25 Pa∙s (Epidian; Ciech Sarzyna, Nowa Sarzyna, Poland), photoinitiator Omnirad 127 (IGM Resin, Waalwijk, The Netherlands), multifunctional monomer Laromer 9023 (BASF, Ludwigshafen, Germany), the Lewis acid adduct (Nacure Super Catalyst A218; Worleé Chemie, Hamburg, Germany) as latent curing agent, and Byk 4510 (Byk-Chemie, Wesel, Germany) as adhesion promoter.

### 2.2. Synthesis of Acrylic Syrups

**Figure 1:**
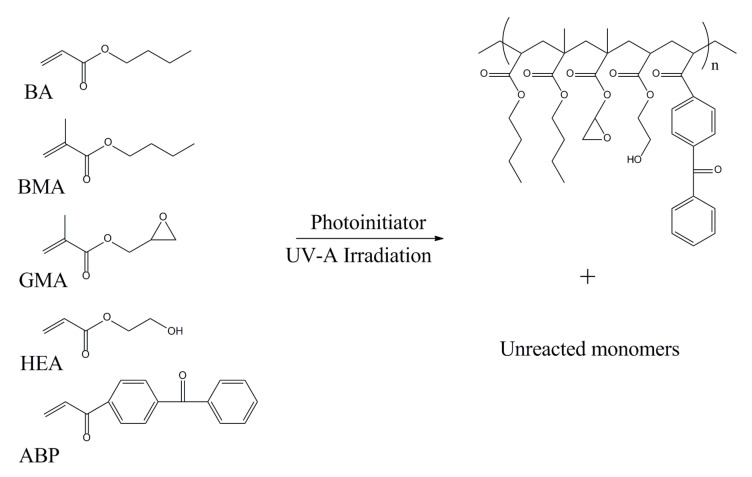
Chemical structures of monomers and synthesis of acrylic syrups.

The acrylic syrups (ASs) were prepared via free-radical bulk-photopolymerization process (FRBP) of BA (6 mol), BMA (2 mol), GMA (1 mol), HEA (1 mol), and ABP (0.1 mol) using 0.1 or 0.2 mol of photoinitiator (HPs/APOs or blends). Choice of monomers results from the work carried out previously by the authors. Each of the monomers plays a role in the system, i.e., BA, self-adhesive properties; BMA positively influences the FRBP process and increases the glass transition temperature of the copolymers; GMA and HEA take part in the thermal curing reaction with epoxy resin; and ABP, copolymerizing photoinitiator, takes part in the formation of cross-linked poly(meth)acrylate network (at the stage of preparing the SAT). Chemical structures of monomers’ and synthesizers’ copolymer chain are shown in Figure 1. The copolymerization processes were realized at 20 °C for 60 or 120 min in a glass reactor (250 mL), equipped with a mechanical stirrer and thermocouple, and in the presence of argon as an inert gas. A mixture of monomers (50 g) was introduced into the reactor and purged with argon for 20 min. The high-intensity UV lamp (UVAHAND 250, Dr. Hönle AG UV Technology, Gräfelfing, Germany) as a UV radiation source was used and it was placed perpendicularly to the side wall of the reactor. The UV irradiation inside the reactor (10 mW/cm^2^) was controlled with UV-radiometer SL2W (UV-Design, Brachttal, Germany). The reactor was water-cooled. The reaction was carried out for 60 min.

Nevertheless, if monomer conversion was <<0.1 Pa·s after 60 min, the reaction was repeated and conducted for 120 min with twice the amount of photoinitiator. Compositions of ASs are presented in Table 2.

#### Characterization of the Acrylic Syrups

Dynamic viscosity of the ASs was measured at 23 °C by means of DV-II Pro Extra viscometer (spindle #6 or #7, 50 rpm; Brookfield, New York, NY, USA). Solid content in ASs after FRBP process was determined using Moisture Analyzer MA 50/1.X2.IC.A (Radwag, Radom, Poland). Samples (ca. 2 mg) were heated in aluminum scale pans at the temperature of 105 °C for 4h.

Monomers’ conversion after FRBP process was determined by proton nuclear magnetic resonance ^1^H NMR (Bruker DPX Avance III HD Spectrometer; 400 MHz). Naphthalene as internal standard was used, and samples of ASs were dissolved in CDCl_3_. The conversion of monomers were determined by comparing the intensity of monomer peaks at 5.81 ppm (BA), 5.55 ppm (BMA), 5.61 ppm (GMA), 5.88 ppm (HEA), and 6.65 ppm (ABP) against the intensity of peaks of the internal standard at 7.5 ppm and 7.8 ppm (naphthalene). The solid content in acrylic syrups was determined using the thermogravimetric method (105 °C/4 h; moisture analyzer Radwag MA 50.R, Radwag, Radom, Poland).

Gel permeation chromatography (GPC) was used to determine molecular masses (*M_w_*, *M_n_*) and polydispersity (PDI) of the copolymers (acrylic syrups were dried at 140 °C for 4 h before the test to remove unreacted monomers). The GPC apparatus contained the refractive index detector (Merck Lachrom RI L-7490), pump (Merck Hitachi Liquid Chromatography L-7100), and interface (Merck Hitachi Liquid Chromatography D-7000) and the Shodex OHpak SB-806M MQ column with Shodex OHpak SB-G precolumn. The GPC tests were performed using polystyrene standards (Fluka and Polymer Standards Service GmbH, Mainz, Germany) and tetrahydrofurane.

### 2.3. Preparation and Characterization of Self-Adhesive Structural Tapes (SATs) and Al/SAT/Al Joints

**Figure 2:**
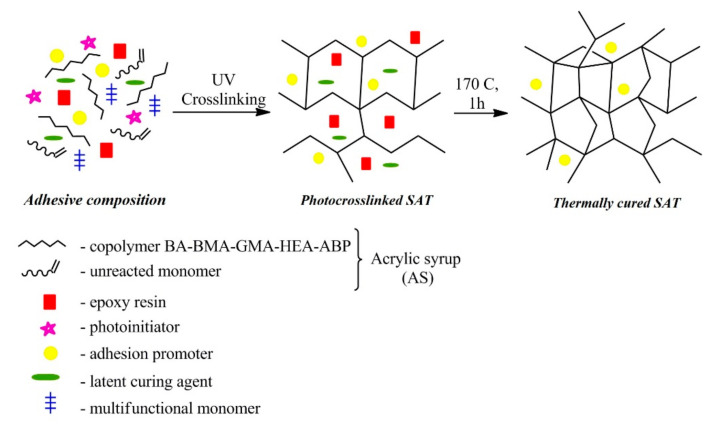
Preparation steps of the self-adhesive structural tapes (SATs).

The SATs were compounded using the AS (50 wt parts), the epoxy resin (50 wt parts), the latent curing agent (1.5 wt part), multifunctional monomer (2 wt part), photoinitiator Omnirad 127 (3 wt part), and adhesion promoter (0.75 wt part). The addition of multifunctional monomer and photoinitiator was to increase the cross-link density of the system. The preparing steps of SATs are shown in Figure 2.

The compositions were applied onto polyester foils (samples for self-adhesive tests) or siliconized paper (other tests) and UV irradiated for 30 s (8 J/cm^2^) using the medium pressure mercury lamp (UV-ABC; Hönle UV-Technology, Gräfelfing, Germany). The UV exposition was controlled with the radiometer (Dynachem 500; Dynachem Corp., Westville, IL, USA). Thickness of the UV-photo cross-linked SATs’ layers were 120 µm. Self-adhesive properties of the thermally uncured SATs were tested according to AFERA 4001 (adhesion to a steel substrate) and AFERA 4015 (tack).These parameters were evaluated using three samples of each adhesive tape. Differential scanning calorimetry (DSC Q100, TA Instruments, New Castle, DE, USA) was used for determination of the glass transition temperature (*T*_g_) of the SATs, enthalpy of SAT curing processes (Δ*H*), onset temperature of the curing reactions (*T*_i_), and maximum temperature of the curing reaction (*T*_p_). Samples (ca. 10 mg) were analyzed using standard aluminum pans at the temperature range of −80 to 350 °C (heating rate of 10 °C/min). Two DSC measurements for each composition were carried out.

Aluminum–SAT–aluminum overlap joints (Al/SAT/Al) were prepared using the SATs and degreased 2024 aluminum panels (100 × 25 × 1.6 mm). The joints were thermally cured at 170 °C for 60 min. Shear strength of the Al/SAT/Al systems was measured at room temperature according to the ASTM D1002-10 standard (10 samples of each system) using the Z010 machine (Zwick/Roell, Ulm, Germany). Cross-linking degree (α) of thermally cured SATs was calculated using DSC data according to Equation (1) [39]

(1) $$\alpha =\left(\frac{\Delta H_{T}-\Delta H_{\mathrm{res}}}{\Delta H_{T}}\right)\;(a.u.)$$

where Δ*H_T_* is the total enthalpy of the SAT curing process (J/g) and Δ*H*_res_ is the enthalpy of a postcuring process of the thermally cured SAT (in a Al/SAT/Al joint).

Dynamic mechanical analysis (DMA) was performed by using the DMA Q800 (TA Instruments, USA). The testing configuration was the dual cantilever mode, with the nominal sample dimension of 50 × 10 × 2 mm and a heating ramp of 3 °C/min. All tests were performed by setting 20 μm as the amplitude and 1 Hz as the frequency. For thermal (DSC) and thermo-mechanical (DMA) properties, three tests for each sample were performed.

## 3. Results

### 3.1. Properties of the Acrylic Syrups

Selected physico-chemical features (i.e., viscosity, solid content, and molecular masses) of the ASs are presented in Table 3. As can been seen, four syrups (i.e., AS-127/1, AS-184/1, AS-TPO/1, and AS-2022/1) exhibited markedly low viscosity (<<0.1 Pa), which is insufficient due to further applications. PleaThree of these syrups was obtained using HP-type photoinitiators (Omnirad 127, Omnirad 184 and Omnirad 2022; the last one contains about 75 wt.% of α-hydroxyalkylphenone Darocure 1173). It should be noted that ASs obtained using higher dose (2 mol %) of HPs (i.e., AS-127/2, AS-184/2) also exhibited low viscosity value (1 and 1.5 Pa·s, respectively). Interestingly, the viscosity of the sample with a blend of PIs (AS-2022/2, where weight ratio of HP to APO was 70/30) was only 0.1 Pa·s. The viscosity measurements’ results correlated with the solid content test. As can be seen in Table 3, the solid content values for ASs with HP-type photoinitators were lower than others (45 and 49 wt.% for AS-127/2 and AS-184/2, respectively), whereas AS-2022/2 sample reached only 20 wt.% of the analyzed parameter. It should be noted that the viscosity and solid content values correlated with GPC measurements’ data. The AS-127/2 and AS-184/2 samples with almost the same viscosity and solid content values exhibited very similar results of *M_n_* (ca. 13,000 g/mol), *M_w_* (47,000 g/mol), and PDI (3.5 a.u.). The lowest values of molecular weights were detected for AS with Omnirad 2022 (blend of HPs and APO) and *M_n_* amounted to 4800 g/mol and *M_w_* to 22,100 g/mol. Generally, the performed tests proved the adverse effect of photoinitiator mixtures (especially HPs with APO) on the rheological properties of syrups. As can be seen in Table 3, the ASs prepared using APO-type photoinitiators exhibited higher viscosity and solid content than samples with HPs. The viscosity and solid content for AS-TPO/2 were markedly higher (33 Pa·s and 73 wt.%, respectively), but to prepare this syrup 2 mol % of PI was used and UV irradiation time was 120 min. Nevertheless, the molecular weights values were similar as those for the sample with HPs. Higher results of *M_n_* and *M_w_* values were obtained in the case of the sample with Omnirad 819 (16,800 g/mol; 55,100 g/mol) and Omnirad 2100 (20,000 g/mol; 67,500 g/mol). This effect was probably due to the generation of four radicals per Omnirad 819 molecule [40]. Also, the system (AS-2100) containing the mixture of photoinitiators was multi-radical. For this reason, higher molecular weights were obtained. It should be emphasized that all copolymers (prepared from ASs) did not have a unimodal molecular weight distribution. The PDI values amounted to ca. 3.5 a.u., whereas in the case of the sample with the blend of HPs and APO (AS-2022) this value was even higher, i.e., 4.61 a.u.

It has long been noted that the photoinitiator efficiency varies depending on the formulation (composition with monomers) and a photoinitiator can be more efficient in one system but not in another [41]. Moreover, the matching of the PIs’ absorption spectrum with emission spectrum of the light source is crucial [42]. It is known that APO-type photoinitiators (especially Omnirad TPO and Omnirad 819) strongly absorb in the UV-A region (λ_max_ ranging between 365 and 416 nm) [34] and HP-type PIs can be activated by lamps emitting up to 380 nm [40]. The UV lamp used for testing emits only UV-A radiation (320–390 nm), so matching the tested PIs to UV source seems to be correct. As we mentioned, there are no photoinitiators dedicated to mass/bulk-photopolymerization processes (FRBP). However, among the products available on the market, APOs proved to be appropriate because they are excellent at facilitating depth cure in coatings. The researchers revealed that the number of radicals generated from the photoinitiator is of great importance in the FRBP process. Photoinitiators generating more than two kinds of radicals (like Omnirad 819 and Omnirad 2100) have been found to be more suitable for FRBP process because they allow syrups with higher viscosities and molecular weights to be obtained.

**Figure 3:**
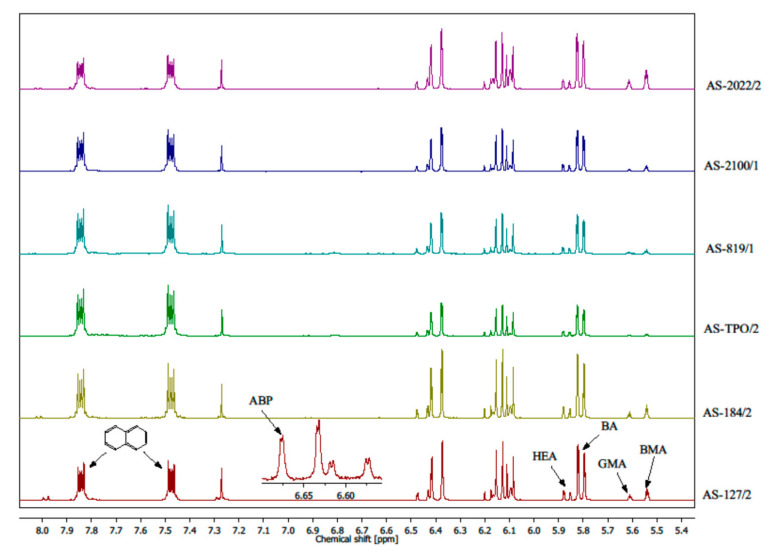
Stacked plot of the proton nuclear magnetic resonance (^1^H NMR) spectra of acrylic syrups. Peaks monitored for each monomer and the internal standard (naphthalene) are indicated.

The influence of PIs’ type on the monomer conversion in multi-component systems has not been widely described in the literature. The present research on ASs’ preparation should be considered as pioneering in this field. In Figure 3, the stacked plots of the ^1^H NMR spectra of acrylic syrups are presented.

The monomers’ conversion (C- total conversion and conversion of individual monomers) are presented in Table 4.

As can be seen, the total monomers’ conversion values (C) are similar to those determined by thermogravimetric method (solid content values in Table 3). The highest results exhibited AS-TPO/2 (75%) and AS-819/1 (68%). As the conversion of an individual monomer is concerned, it can be seen that very high conversion of BMA and GMA was achieved in systems with APO-type PIs. BMA conversion amounted to 94% (using Omnirad TPO), 90% (Omnirad 819), and 85% (Omnirad 2100) and GMA conversion reached 95, 90, and 89% (in the same order). The conversion of ABP was very high as well (79–89%). Generally, in all syrups the conversion of methacrylate monomers (BMA and GMA) was almost two-fold higher than that of acrylate monomers (BA and HEA). NMR studies have shown that ASs consisted of copolymer part and unreacted monomers’ mixture, mainly BA and HEA. According to NMR measurements, data could be calculated (not presented) such that ASs with APO-type PIs (AS-TPO/2, AS-819/1, and AS-2100/1) contained a copolymer BA-BMA-GMA-HEA-APB (in which the molar ratio of monomers was ca. 4/1.9/1.9/0.7/0.09, respectively) and mixtures of unreacted BA (ca. 2 mol) and HEA (ca. 0.3 mol). In contrast, acrylic syrups based on HP-type PIs and HPs/APO blend (AS-Omnirad 2022), in addition to unreacted BA and HEA, contained a lot of BMA and GMA as well.

### 3.2. Properties of Thermally Uncured SATs Based on Acrylic Syrups

**Figure 4:**
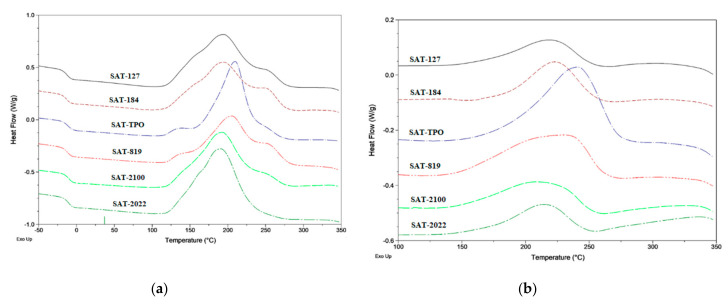
Differential scanning calorimetry (DSC) thermographs for the uncured SATs (**a**) and cured SATs (**b**) based on ASs.

Based on the prepared ASs and the commercial epoxy resin (ER), the thermally curable pressure-sensitive adhesive films (SATs) were prepared. In this paper, we are presenting a new, solvent-free method of obtaining the SATs, i.e., preparing the adhesive binder (epoxyacrylate copolymer) via FRBP process. Thermal curing process of the SATs (based on the ASs’ components) are presented in Table 5 and Figure 4. As can be seen, the SATs exhibited one *T*_g_ value. This confirmed that the ingredients (ER and methacrylate copolymers) were thermodynamically miscible. The *T*_g_ values were quite similar (−10 °C, −11 °C, or −12 °C), but it should be emphasized that SATs received using the HP-type PIs (SAT-127, SAT-184, and SAT-2022) characterized a bit lower *T*_g_ value (−12 °C) than others. Interestingly, the mentioned SATs exhibited markedly higher enthalpy of cationic curing process of epoxy groups (Δ*H*) (247 J/g, 233 J/g, and 253 J/g, respectively) as well as lower onset temperature of the process (*T*_i_) (120 °C, 135 °C, and 136 °C, respectively). Arguably, this effect was related to the properties of the acrylic syrups. AS-127, AS-184, and AS-2022 were characterized by lower monomer conversion (23–52%, Table 4) than ASs with APO-type PIs (62–75%) as well as lower molecular masses (Table 3). As we stated in this paper, AS-127, AS-184, and AS-2022 syrups contained few low-molecular copolymers of BA-BMA-GMA-HEA-ABP and all types of unreacted monomers.

Probably, on the SAT preparation stage, during UV irradiation in the presence of ER and other ingredients from unreacted monomers, low-molecular products (methacrylate copolymers) were formed. Instead, the process of polymerization of epoxy groups (at the stage of aluminum joints’ preparation; the curing process in high temperature) was easier (lower *T*_i_ and higher Δ*H*) in the presence of low-molecular copolymers (formed at the stage of preparing the adhesive film). The opposite effect was in the case of SAT with APO-type PIs. The characteristic temperatures of cationic polymerization of epoxy groups (*T*_i_ and *T*_p_) were higher, i.e., 167 °C and 210 °C (for SAT-TPO) and 149 °C and 206 °C (for SAT-819). However, enthalpy values were lower (209 J/g for SAT-TPO and 223 J/g for SAT-819). An additional confirmation of this effect of syrups was the result of the cross-linking degree (α, Table 5). Cross-linking degree measurements (DSC) were performed for thermally cured SATs. Generally, a higher α value was demonstrated for SAT received using the HP-type PIs (from 0.81 to 0.88 a.u.) than SAT with APOs (0.50 for SAT-TPO and 0.67 for SAT-819). The highest values of α were achieved for SAT-2022 (0.88) (HPs/APO blend) received from AS-2022/2 with the lower value of monomers’ conversion (23%, Table 4).

**Figure 5:**
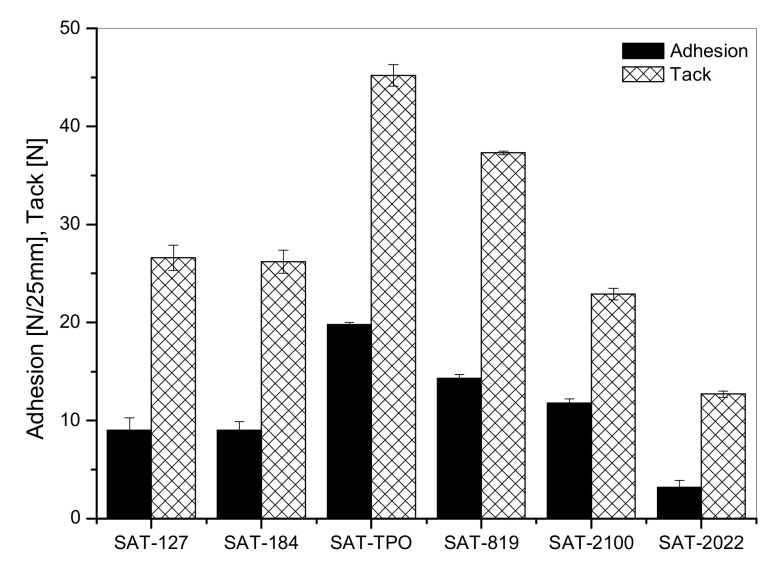
Adhesion to steel and tack for thermally uncured SATs based on ASs.

The thermally uncured SATs were characterized in terms of their basic self-adhesive features (i.e., adhesion to a steel and tack). Results are presented in Figure 5. As can be seen, SATs based on APO-type PIs exhibited significantly higher values of adhesion (20 N/25 mm for SAT-TPO; 14 N/25 mm for SAT-819 and 12 N/25 mm for SAT-2100) and tack (45 N, 37 N and 23 N, respectively). On the other hand, the lowest value of features under discussion was noticed for SAT-2022 (3.2 N/25mm and 12.7 N). It should be noted that SAT-TPO, SAT-819, and SAT-2100 were based on ASs that were characterized by high monomers’ conversion (C) and high viscosities compared to ASs with HP-type PIs. Considering the SATs based on APO photoinitiators, it can be seen that adhesion values decreased with increasing *M_n_* and *M_w_* values in ASs. This was accordance with the literature reports on the effect of molecular weight on adhesion features [43]. However, this interpretation of the effect of photoinitiators on adhesion can only be used in systems with high and similar monomers’ conversion. It should be noted that SAT films were prepared on the way of UV irradiation of the mixtures of ASs (with unreacted monomers) and ER (mainly). The aspect of amount of unreacted monomers’ mixtures seems to be crucial. The high content of unreacted monomers in the AS/ER system intended for UV cross-linking reduced the adhesive properties (adhesion to a steel and tack) of obtained SAT.

### 3.3. Properties of Thermally Cured SATs Based on Acrylic Syrups

**Figure 6:**
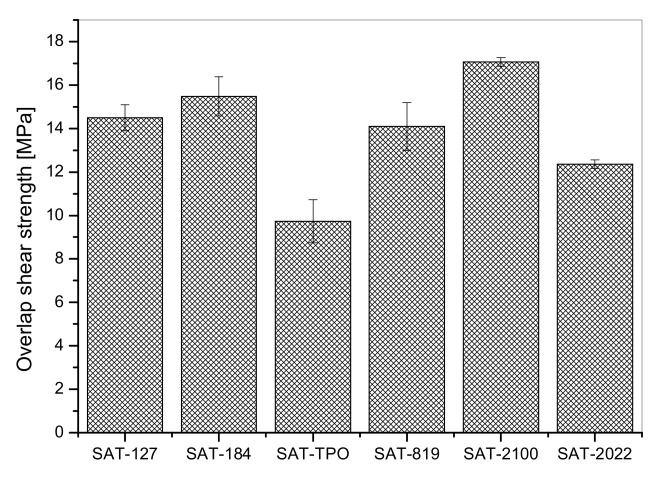
Shear strength for thermally cured aluminum–SAT–aluminum overlap joints.

**Figure 7:**
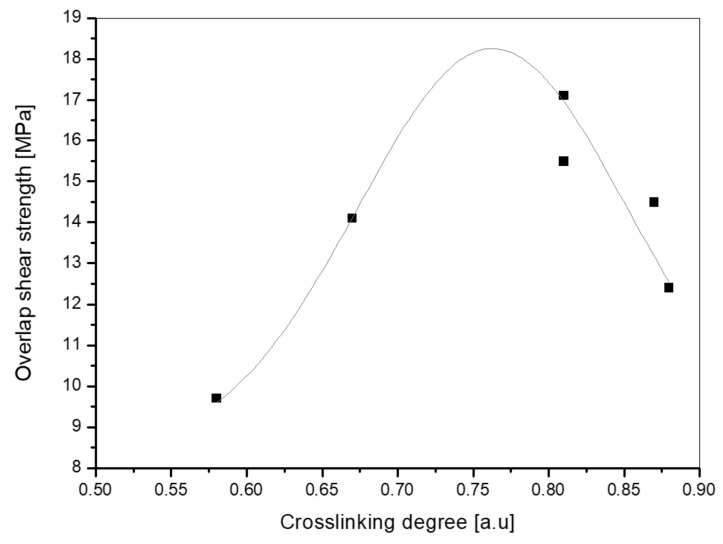
Influence of cross-linking degree (α, DSC measurements) of thermally cured SATs on overlap shear strength for aluminum–SAT–aluminum overlap joints.

The SATs were applied onto 2024 T3 aluminum panels and the prepared Al/SAT/Al overlap joints were thermally cured at 170 °C for 60 min. Shear strength (τ) values recorded for the cured joint are presented in Figure 6. As can be observed, the best result (17.1 MPa) was noted for Al/SAT-2100/Al. Joints with SAT-127 and SAT-184 also exhibited high results (14.5 MPa and 15.5 MPa, respectively). Interestingly, the Al/SAT-TPO/Al sample reached the lowest value (9.7 MPa) of the parameter. On this basis, it can be concluded that shear strength of SAT films does not depend on adhesion (adhesion of SAT-TPO was the highest and reached 20 N/25 mm). In Figure 7 the results of shear strength and cross-linking degree (α, DSC measurements of cured SATs, Table 5) are summarized. As it turned out, there was a certain “optimal” cross-linking degree of adhesive at which the adhesive joint exhibited the highest shear strength.

In the case of SAT products, it was about 0.7 to 0.8 a.u. The presented results of the influence of cross-linking density on shear strength are consistent with the literature reports [44] and our previous unpublished research.

**Figure 8:**
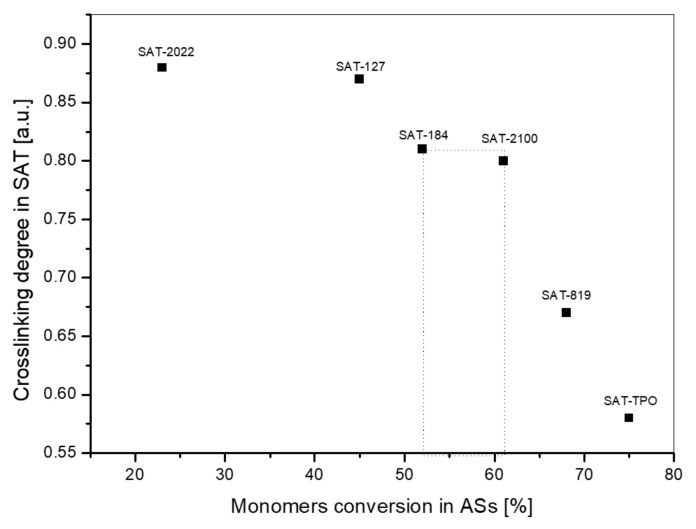
Influence of the monomers’ conversion of ASs (C, NMR data) on cross-linking degree (calculated using DSC data) for thermally cured SATs.

In the next step, we compared the results of cross-linking degree of cured SATs and monomers’ conversion of ASs used for SAT preparation. The results are shown in Figure 8. As can be seen, the cross-linking degree values (α) markedly decreased with increasing the monomers’ conversion in ASs. As we mentioned, the highest result of τ was registered for SAT-2100 (17.1 MPa) and SAT-184 (15.5 MPa) samples, which were characterized by the α values 0.81 and 0.8 (respectively) and total conversion of monomers (C) 62% (SAT-2100) and 52% (SAT-184). We assumed that the monomers’ conversion interval can be indicated by which the cross-linking degree giving the highest shear strength is obtained. Based on the performed research, we claim that syrups with a ca. 50–60% of monomers’ conversion are of great importance here.

**Figure 9:**
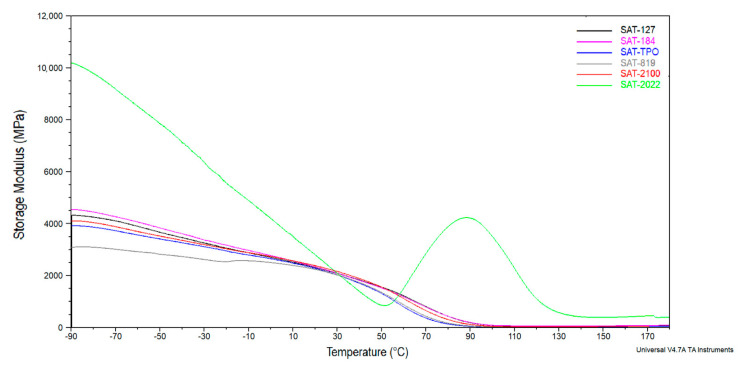
Storage modulus for thermally cured SATs.

**Figure 10:**
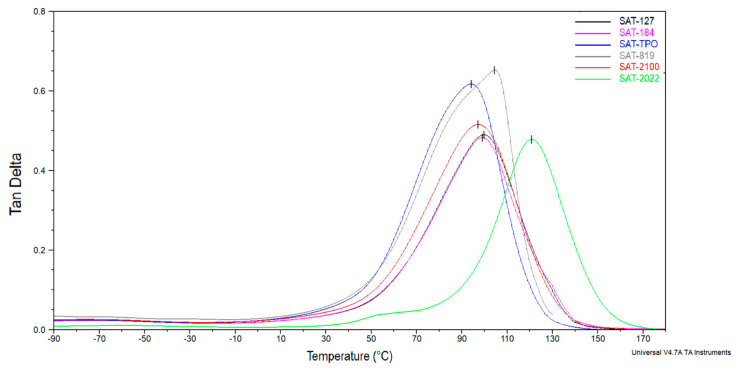
Tangens delta for thermally cured SATs.

The dynamic mechanical properties of SATs based on ASs were examined by DMA in the −90 to 180 °C temperature range. Figure 9 shows the storage modulus (*E*’) and Figure 10 shows the tan δ as a function of temperature. Storage modulus at room temperature and 150 °C and glass transition temperatures for cured SATs are summarized in Table 6.

DMA curves for SATs are similar to those for thermosetting epoxy resins. Notwithstanding, DMA analysis revealed differences between SATs in the glassy region (especially in the range of −90 °C to 0 °C). Probably, the methacrylate copolymers (i.e., ASs) are responsible for maintaining the SATs in the area of low temperatures. It can be seen that all SATs’ samples showed similar behavior except for SAT-2022. In this case, the peak on storage modulus curve can be observed (with initial temperature ca. 50 °C and maximum temperature ca. 89 °C), which means that the additional cross-linking reaction occurred. From the literature, cases of additional curing of weakly cross-linked samples during DMA tests are known [45]. We claim that due to the low monomers’ conversion of AS-2022 (23%, Table 4), the formation of short methacrylate chains during UV irradiation and uncured SAT-2022 preparation was possible, so that the cationic cross-linking process of epoxy groups was not disturbed. Therefore, it is possible to supplementary cross-link during the DMA analysis. Our research revealed that cured SAT-2022 exhibited the higher cross-linking degree (α = 0.88, after curing at 170 °C for 60 min), so the storage modulus at room temperature of this sample was higher (2454 MPa) in comparison to other samples (+7–14%). After additional cross-linking during DMA analysis the value of the storage modulus at 150 °C (383 MPa) was significantly higher (+660–1915%). Additionally, the *T*_g_ value for SAT-2022 was outstanding (120 °C, Table 6). The *T*_g_ values for other SATs were comparable (ca. 100 °C).

## 4. Conclusions

In this paper, the new and environmentally friendly method of obtaining thermally curable structural self-adhesive tapes was presented and the effect of used, different type I photoinitiators on selected features of the obtained acrylic syrups and adhesive and (thermo)mechanical properties of SATs were studied. It was stated that the kind of photoinitiator significantly influenced the physico-chemical features of ASs. More suitable are PIs generating more kinds of radicals. Definitely higher results of viscosity, solid content, monomers’ conversion, and molecular weight in ASs were obtained when APO-type PIs were used, especially bis(2,4,6-trimethylbenzoyl)-phenylphosphineoxide (Omnirad 819) or its mixtures with 2,4,6-trimethylbenzoyl-diphenyl phosphine oxide (Omnirad 2100). Additionally, ASs obtained using APO-type PIs were characterized by higher adhesive properties (adhesion to a steel and tack). It was found that a higher content of unreacted monomers (>50 wt.%) reduced the adhesive properties of SATs after UV cross-linking but increased the cross-linking degree of SATs after thermal curing. In turn, the cross-linking degree had a decisive impact on shear strength of aluminum-SAT-aluminum joints. The optimal value of the mentioned parameter was indicated (ca. 0.7-0.8 a.u.). In order to achieve this ratio, the ASs with a monomers’ conversion of ca. 50–60% should be prepared. Considering the above, the highest shear strength results were obtained with SAT-184 (prepared with 2 mol % of HP-type PI) and SAT-2100 (1 mol % of APO-type PIs). It seems that kind of PIs does not have a crucial impact on mechanical properties of SATs but the appropriate monomers’ conversion is necessary. Nevertheless, the fact is that monomers’ conversion in FRBP process on the level of 50–60% may by already achieved using the 1 mol % of APO-type PIs and, in the case of HP-type PIs, 2 mol % of initiator is needed. Considering the thermo-mechanical properties of cured SATs, it should be mentioned that they basically differed only in the area of low temperatures. On the other hand, the sample obtained from AS with significantly low monomers’ conversion underwent typical postcuring effects during the test and translated into an increase in the glass transition temperature of SAT.

## Figures and Tables

**Table 1:** Structures of tested photoinitiators (PI).

PI Type	PI Names and Chemical Structure	
HPs	Omnirad 127	Omnirad 184
	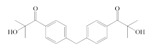	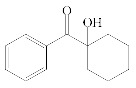
APOs	Omnirad 819	Omnirad TPO
	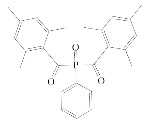	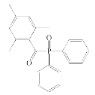
Blends	Omnirad 2100 (O.TPO-L + O.819) (95/5)	Omnirad 2022 (Darocure 1173 + O.819 + O.TPO-L) (75/17.5/7.5)
	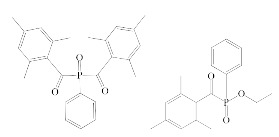	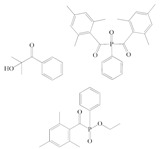

**Table 2:** Compositions of monomers and photoinitiators for preparation of acrylic syrups (ASs).

AS Acronym	Monomers (mol %)					PI	
	BA	BMA	GMA	HEA	ABP	Name	Mol %
AS-127/1	58.8	19.6	9.8	9.8	0.98	Omnirad 127	1
AS-127/2	58.2	19.4	9.7	9.7	1	Omnirad 127	2
AS-184/1	58.8	19.6	9.8	9.8	0.98	Omnirad 184	1
AS-184/2	58.2	19.4	9.7	9.7	1	Omnirad 184	2
AS-TPO/1	58.8	19.6	9.8	9.8	0.98	Omnirad TPO	1
AS-TPO/2	58.2	19.4	9.7	9.7	1	Omnirad TPO	2
AS-819/1	58.8	19.6	9.8	9.8	0.98	Omnirad 819	1
AS-2100/1	58.8	19.6	9.8	9.8	0.98	Omnirad 2100	1
AS-2022/1	58.8	19.6	9.8	9.8	0.98	Omnirad 2022	1
AS-2022/2	58.2	19.4	9.7	9.7	1	Omnirad 2022	2

BA—n-butyl acrylate; BMA—butyl methacrylate; GMA—glycidyl methacrylate; HEA—2-hydroxyethyl acrylate; ABP—4-acryloyloxy benzophenone.

**Table 3:** Dynamic viscosity, solid content, and molecular weights of ASs.

AS Symbol	η (Pa∙s)	Solid Content (wt%)	*M*_n_ (g/mol)	*M*_w_ (g/mol)	PDI
AS-127/1	<0.1	n.d.	n.d.	n.d.	n.d.
AS-127/2	1.0	45	13,170	47,100	3.58
AS-184/1	<0.1	n.d.	n.d.	n.d.	n.d.
AS-184/2	1.5	49	13,850	48,400	3.5
AS-TPO/1	<0.1	n.d.	n.d.	n.d.	n.d.
AS-TPO/2	33	73	13,550	48,100	3.55
AS-819/1	14.0	66	16,800	55,100	3.28
AS-2100/1	6.5	58	20,000	67,500	3.38
AS-2022/1	<0.1	n.d.	n.d.	n.d.	n.d.
AS-2022/2	0.1	20	4800	22,100	4.61

n.d.—no data; η—viscosity; *M*_n_—number average molecular weight; *M*_w_—weight average molecular weight; PDI—polydispersity.

**Table 4:** Monomers’ conversion calculated by proton nuclear magnetic resonance (^1^H NMR) analyses.

AS Symbol	Monomers Conversion (%)					
	C ^1^	BA	BMA	GMA	HEA	ABP
AS-127/2	45	35	66	68	30	65
AS-184/2	52	42	72	73	38	68
AS-TPO/2	75	65	94	95	67	89
AS-819/1	68	57	90	90	57	88
AS-2100/1	62	50	85	86	50	79
AS-2022/2	23	17	38	36	11	37

^1^ C is total monomers’ conversion.

**Table 5:** Thermal features of uncured self-adhesive tapes (SATs) based on ASs and cross-linking degree of cured SATs.

SAT Acronym	*T*_g_ (°C)	*T*_i_ (°C)	*T*_p_ (°C)	Δ*H* (J/g)	α (a.u.)
SAT-127	−12	120	194	247	0.87
SAT-184	−12	135	195	233	0.81
SAT-TPO	−11	167	210	224	0.59
SAT-819	−11	149	206	209	0.67
SAT-2100	−10	138	192	223	0.81
SAT-2022	−12	136	191	253	0.88

*T*_g_—glass transition temperature; *T*_i_—onset temperature of the curing reactions; *T*_p_—maximum temperature of the curing reaction; Δ*H*—enthalpy of SAT curing processes; α—cross-linking degree of thermally cured SATs.

**Table 6:** Characteristic storage modulus (*E*’) values at room temperature and 150 °C and the glass transition temperature (*T*_g_) values for cured SATs based on ASs.

SAT	*E*’_(25 °C)_ (MPa)	*E*’_(150 °C)_ (MPa)	*T*_g_ (°C)
SAT-127	2285	50	100
SAT-184	2216	58	99
SAT-TPO	2136	28	94
SAT-819	2156	20	103
SAT-2100	2202	35	96
SAT-2022	2454	383	120
